# Early food for future health: a randomized controlled trial evaluating the effect of an eHealth intervention aiming to promote healthy food habits from early childhood

**DOI:** 10.1186/s12889-017-4731-8

**Published:** 2017-09-20

**Authors:** Christine Helle, Elisabet Rudjord Hillesund, Mona Linge Omholt, Nina Cecilie Øverby

**Affiliations:** Department of Public Health, Sport and Nutrition, Faculty of Health and Sport Sciences, University of Agder, PO Box 422, 4604 Kristiansand, Norway

**Keywords:** Parental feeding practices, Child eating behavior, Infant, Childhood overweight and obesity

## Abstract

**Background:**

Childhood overweight and obesity is a global public health challenge. Primary prevention initiatives targeting parents have been called for to encourage a positive feeding environment and healthy eating habits that may lay a good foundation for future health. At the same time, there is a need for interventions which combine accessibility and scalability with cost effectiveness.

Today’s parents are extensive Internet-users, but only a few randomized controlled trials have investigated the use of Internet to promote healthy eating habits in early childhood. In *Early Food for Future Health* we have developed and will evaluate an Internet-based tool for parents of children between 6 and 12 months, aiming to increase knowledge about infant nutrition and foster protective feeding behavior.

**Methods:**

During springtime 2016, parents of children aged between 3 and 5 months were recruited through Norwegian child health centres and announcements on Facebook. After completing the baseline questionnaire, 718 parents were individually randomized to intervention- or control group. The intervention group received monthly emails with links to an age-appropriate web-site when their child was between 6 and 12 months. The control group received ordinary care from the child health centres.

The data-collection is ongoing. All participants will be followed up at ages 12 and possibly 24 and 48 months, with questionnaires relating to eating behaviour and feeding practices, food variety and diet quality.

**Discussion:**

Providing guidance and counseling to parents of infants is an important task for health authorities and the public child health services. *Early Food for Future health* is an intervention focusing on promoting early healthy food-habits which may prevent childhood overweight and obesity. If proven to be effective, *Early Food for Future Health* can be used by parents and public health nurses for supplementary guidance on feeding practices and diet.

This study has the potential to provide greater insight and understanding regarding early parental feeding practices, child eating behavior and the development and efficacy of Internet-based public health interventions.

**Trial registration:**

ISRCTN13601567.

## Background

Childhood overweight and obesity represents a huge public health challenge globally [1, 2]. Childhood overweight and obesity entails major negative consequences for children’s physical and mental health, and track into adulthood with attendant metabolic derangements that increase morbidity and mortality [3, 4]. There is also evidence of a relationship between early unhealthy dietary patterns and poorer mental health in children and adolescents [5]. In Norway, childhood overweight and obesity are affecting 11–16% of the children, and there is a trend towards higher prevalence of childhood overweight and obesity in the group of children with parents of lower socioeconomic status [6].

The early feeding environment is critical for establishing eating habits that may influence weight development and healthy growth in the long term [7, 8]. Todays’ parents are raising their children in an obesogenic environment characterized by high access to energy-dense non-core foods [9]. Rapid early weight gain during the first 2 years is strongly associated with adiposity in later childhood and adolescence [10]. Since parents are gate-keepers of food and contribute to their child’s eating in numerous ways, primary intervention initiatives targeting parents have been called for with the aim of encouraging and facilitating a positive feeding environment for the development of healthy eating habits [11].

Throughout the weaning period, the infant’s diet changes dramatically from the initial exclusive milk diet to a diet resembling that of an adult’s. This requires constant adaptation and adjustment of parental feeding behaviors as the child’s development of eating skills progresses. It is vital for parents as a part of the parenting process to understand their children’s developmental stages and physical and psychological needs. Although parental guidance on infant food and early nutrition is an important and prioritized task at the Norwegian public child health centers [12], many parents feel insecure about their own skills and expertise and wishes more information on this field [13].

Barriers to fostering healthy eating habits in children include parental feeding practices that prevent the child from using hunger and satiety signals to initiate and stop eating [14]. This may undermine the innate capacity to self-regulate energy intake which develops during infancy. Controlling feeding practises such as pressure and restriction [14], as well as the emotional use of food as reward or to calm, may also undermine children’s capacity to self-regulate [15]. Other influential early feeding practices are strategies to increase dietary variation facilitating acceptance for healthy foods [16], like repeated exposures to new foods in order to overcome infant rejection of healthy foods such as vegetables.

So far only a few randomized controlled trials have investigated methods of primary prevention of obesity commencing during infancy [8, 17–19], the largest being the Australian NOURISH study which confirms that maternal feeding practices are modifiable through early intervention with anticipatory guidance on “when, what, and how” of infant feeding [17]. This randomized controlled intervention study targeted early feeding practices through educational group sessions in first-time Australian parents. The intervention resulted in increased use of protective feeding practices in the intervention group, with higher reported levels of responsive feeding and lower levels of non-responsive feeding practices [17]. These intervention effects were maintained more than 3 years after intervention completion [20].

The prevention of overweight and obesity in children and adolescents is an important issue in public health. To develop public health interventions which combine easy accessibility with cost effectiveness, is therefore of great importance. Several researchers have suggested that Internet-based interventions should be developed and evaluated to inform the development of health promotion programs to be used in primary care. An Italian study from 2013 showed that 95% of pregnant women were eHealth seekers [21]. We assume that the same numbers hold when they move on to nourish their child, and that the use of eHealth in health promotion initiatives could be a relevant alternative when targeting parents [22].

The difference between separate socioeconomic groups, makes it particularly important that interventions are adapted for the group of parents from lower socioeconomic backgrounds. The Internet availability is steadily increasing, and parents from less privileged socio-economic groups are among the most prevalent users of Internet-based parental sites for support and information [23]. Online interventions may offer versatile parenting support in the form of information, peer, and professional support [24]. Salonen et al. (2013) investigated the impact of an Internet-based intervention offering online support for parenting, breast feeding and infant care [25]. They experienced that young mothers perceived the Internet resource to be more beneficial than older mothers, and that the longer the period they used on the Internet resource per week, the more positive the perception they had about its usefulness. However, a large proportion of parents find it difficult to assess the quality of web-based information and to find the information they seek [26]. Bert et al. (2013) state that to reduce the likelihood for women of finding erroneous information and misinterpreting correct ones, health care professionals should be committed to filling the information gap and guide women in their online searches [21].

*Early Food for Future Health* is such a commitment. To our knowledge only few previous studies have investigated the utility and effect of Internet-based interventions on these topics, which might suit the new parent-generation to a larger extent than usual child care [27]. This project targets the following societal challenges; the growing obesity epidemic, the burden of non-communicable diseases, and the shift in society towards primary health care and preventive medicine. Through *Early Food for Future Health* we have developed and will evaluate an Internet-based tool to be implemented in the primary care of small children that, if proven to be effective, can be used by parents and public health nurses for supplementary guidance on feeding practices and diet.

### Rationale for the intervention content

*Early Food for Future health* is a primary prevention intervention targeting parental behavior. There is substantial causal evidence for parenting affecting child eating behaviour in early life [28]. Schwartz et al. [29] suggest that new guidelines should especially help parents to establish a responsive feeding behaviour. This is in line with contemporary research on the field. According to a systematic review from 2015, the most promising obesity prevention interventions for children under 2 years of age are those that focus on diet and responsive feeding [19]. The NOURISH study highlighted the importance of protective complementary feeding practices to promote self-regulation of intake and development of healthy food preferences [20]. Daniels et al. used two underpinning themes in their intervention: Theme 1: repeated neutral exposure to unfamiliar foods and limiting exposure to unhealthy foods to promote the development of healthy food preferences. Theme 2: responsive feeding that recognises and responds appropriately to infant cues of hunger and satiety to maintain infants’ innate capacity to self-regulate intake and avoid overfeeding [30]. Both themes are included and emphasized also in *Early Food for Future Health*.

General parenting styles reflect the prevailing emotional climate provided by the parents, and are regarded as relatively stable traits that are consistent across time and context [31, 32]. Parental practices refer to what parents do in a given context, like feeding practices during mealtimes [33]. The concept of ‘food parenting’ comprises parents’ knowledge, beliefs, affects, and behaviors towards their children and food [11]. All aspects of food parenting need to be addressed to beneficially influence childhood diet [7]. In *Early Food for Future Health*, we seek to provide parents with anticipatory guidance on *when, what* and *how* on three levels:

1. Promote accurate knowledge and skills about child nutrition and eating-development *(when, what)*
2. Convey knowledge on *how* to promote an early healthy diet and early beneficial eating habits
3. On an overarching level, emphasize a general parenting style that is sensitive to the child’s cues and needs

To promote knowledge about infant nutrition and facilitate sensitive feeding behavior, *Early Food for Future Health* draws upon elements from attachment-theory, social cognitive theory and the framework of anticipatory guidance.

### Attachment-theory

Attachment-theory provide a well-adapted framework to promote sensitive parenting, and was also used in the NOURISH study [7]. Attachment refers to the emotional tie between a child and his/her primary caregiver. Attachment patterns is shaped through repeated interactions between child and caregiver and impact children’s developmental trajectories. According to attachment theory [34, 35], sensitive parenting during early childhood is predictive of child security of attachment and superior child outcomes across a range of domains [36]. In order to form a secure attachment pattern between child and caregiver, the caregiver’s recognition and responsiveness to the infant’s cues is central. Through sensitive interplay, the caregiver provides the child with both a secure base for exploration and a safe haven for emotional regulation of distress. Parental recognition and regulation of the infant’s cues of hunger and satiety is central to sensitive feeding practices. A positive parent-child relationship characterized by high level of responsiveness have been associated with lower child weight and healthier eating [37]. Videotapes of interactions between parent and child are commonly used to improve attachment security [38, 39]. In this study, we use videotapes of parent-child interactions in the feeding situation as part of the intervention.

### Social cognitive theory

Social cognitive theory [40] has been widely used in health education and health promotion interventions, also in multi-component interventions to prevent childhood obesity [17, 27, 41]. Social cognitive theory addresses the interaction between person, environment and behavior. On the personal domain, the videotapes in the intervention will provide participants with knowledge and skills regarding infant nutrition and cooking. On the environmental domain, videotapes of sensitive parent-child interplay during mealtime situations may improve parental feeding practices through social modelling and observational learning. This may lead to increased self-efficacy and enhanced behavioral capability when it comes to nutritional choices, preparing homemade baby food and beneficial feeding practices.

### Anticipatory guidance

Anticipatory guidance is acknowledged as an important aspect of pediatric practice. It provides parents with information during the period just prior to when the developmental aspect will be relevant. By recognizing risk factors early in a child’s life, primary care providers can help families make positive changes that will improve a child’s weight trajectory [42]. This method is previously included in other interventions aiming to prevent childhood obesity [7, 43].

### Objectives and outcomes

The objective of the present study is to develop, implement and evaluate the effect of an eHealth intervention - “barnE-mat” (infant foods) - aiming to promote an early healthy and sustainable [44] diet and healthy food habits in children through encouraging beneficial parental feeding practices and enhancing parental self-efficacy regarding knowledge, skills, and confidence with respect to child feeding. *Early Food for Future Health* is a universal primary prevention intervention irrespective of maternal- or child obesity risk, and provides anticipatory guidance to parents on complementary feeding themes when the child’s age is between 6 and 12 months.

### Primary outcomes

#### Infant primary outcome measures

Child eating behavior; assessed at baseline and after the intervention.

Food intake and food variance; assessed at baseline and after the intervention.

#### Parent primary outcome measures:

Feeding style and feeding practices; assessed at baseline and after the intervention.

Feeding self-efficacy; assessed at baseline and after the intervention.

Parenting style; assessed at baseline and after the intervention.

Making more homemade baby food in the weaning period; assessed after the intervention.

### Secondary outcomes

Child body mass index and weight; assessed before and after the intervention.

### Other study variables

We have included background variables known to be associated with child nutrition, eating habits and development: Child temperament/behavior/sleep, parental socioeconomic background/education, parental food intake, parental eating behavior and parental mental and somatic health.

## Methods/Design

### Study design

The study uses a randomized controlled design to assess the effectiveness of the intervention. The recruitment started in March 2016 and ended in June 2016. Parents from all over Norway (mothers and fathers) were eligible to participate in the study if they had a 3–5 months old child that was born at term with birth-weight ≥ 2500 g, were responsible for providing food to their child, and were literate in Norwegian.

**Fig. 1:**
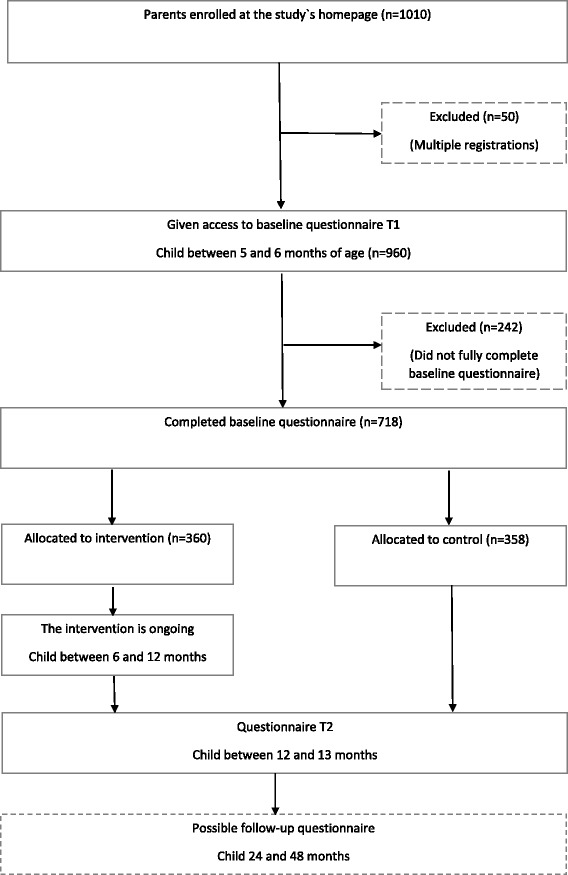
Flowchart of the study design

When completing the baseline questionnaire, the participants were randomly, consecutively and individually allocated to either the intervention group or control group based on a computer-generated list (SPSS), see Fig. 1.

Parents of children randomised to the intervention group were given electronic access to the website and received monthly information on *when*, *what* and *how* to feed their child throughout the weaning period. These emails with links to the age-appropriate web-site, were programmed to be automatically sent from the system based on the infant’s birthdate. The control group received ordinary care from the child health centre. Comparisons between intervention and control group will be done according to the study-outcomes.

After baseline, all participants will be followed up at ages 12 and possibly 24 and 48 months with questionnaires relating to eating behaviour and feeding practices, food variety and diet quality. Information on growth parameters will be requested at the same time-points based on scheduled measurements from routine follow-up at the child health centre.

### Recruitment and participants

We used two parallel recruitment strategies in this study:

An email was sent to all the municipality child health centres in Norway. This email contained a link to a customized information-website for the municipality’s child-health services. The website included an article about the study published in the Norwegian journal for public child health nurses [45] and a short promotional film in addition to printable posters and parent-leaflets. Nurses could, if they found it appropriate, inform their parents about the possibility of participating in this study. Parents who were interested could give their informed consent and enrol themselves on the study’s homepage [46] if their children met the inclusion criteria.

Recruitment was also done through announcement on Facebook. An advertisement with a short film presented the study. Parents wishing to participate could give their informed consent and enrol themselves on the study’s homepage if their children met the inclusion criteria.

The recruitment lasted 3 months, from March to June 2016. During this period 1010 participants enrolled themselves on the study’s homepage, but 50 of the participants registered themselves more than once. Of the remaining 960 participants, 718 parents completed the baseline questionnaire. Of these, the majority were recruited through Facebook (Table 1). Among the participants, there was a very high percentage of mothers compared to fathers, and the group as a whole had a relatively high educational level. The geographical distribution of participants reflected the geographical population density in Norway, except for a slightly higher proportion of participants from the southern parts of Norway and somewhat lower from the eastern parts of Norway.

**Table 1:** Characteristics of the participating parents (*n* = 718)

Characteristics			Value^a^
Recruitment strategy			
		Recruited through Facebook	89,8
		Recruited through child health centers	2,1
		Recruited through both Facebook and child Health centers	2,2
		Other (parents/friends/media)	5,8
Parents			
	Mothers/Fathers		99,6/0,4
	Age (years)		30.5 (4.4)
	Education		
		Upper secondary school or less	19,1
		College/university ( ≤ 4 years)	36.9
		College/university ( ≥ 4 years)	43.9
	Geographic residence		
		Northern parts of Norway	7,5
		Middle parts of Norway	8,5
		Western parts of Norway	29,6
		Southern parts of Norway	14,2
		Eastern parts of Norway (included capital)	40,2

^a^Percentages for categorical variables, means with SD for continuous variables

### Overview of the intervention content

The Internet-based intervention includes seven monthly theme-films of 3 to 5 min’ duration. These monthly videos follow the child’s eating development and are focusing on important developmental aspects and food events at the given ages. They include information on appropriate foods, food texture, tastes of food, portion sizes, how to feed your child, the importance of repeated exposures, how and when the child may feed itself and beneficial parent-child interplay. The intervention also includes monthly access to new recipes and cooking-films that are demonstrating how to make age-appropriate, homemade baby food made of easily available ingredients in an uncomplicated way. The cooking videos are aligned with the themes of the main videos. Every month, a new age-adjusted website is being launched, showing the theme-film of the month together with the corresponding recipes and cooking films.

In the intervention, themes listed in Table 2 are addressed. According to the review by Schwartz et al., these are the most important complementary feeding themes in order to develop healthy eating habits early in life [47]. The themes in Table 2 also include the main potential obesity prevention approaches suggested by Birch and Ventura [48].

**Table 2:** Description of the content in the intervention “barnE-mat”

Age Title/Main theme	Anticipatory guidance on *when* and *what*	Anticipatory guidance on *how*	Intervention-elements based on attachment theory/developmental psychology	Guidance/demonstrating on skills in the movies (examples)
6 months The first food	Time to introduce solid food Small tastings of finely mashed consistency to raise interest Variation in food from the beginning	Guidance of responsive feeding Following the child’s signals of hunger and satiety	Child-centered care; emphasizing the importance of being sensitive and responding to the infant’s cues Confirm and meet the child’s signals with facial expressions and voice	Sensitive/Responsive feeding; mother awaiting her baby’s signals in the meal How to make food tastes and small portions of home-made infant food
7 months Sweet and salty food	Development of taste and food preferences Sensitive period for introduction of new flavors	Use of repeated exposures to promote taste and texture acceptance Avoid pressuring to eat The importance of being a role-model during meals Promoting good mealtime routines	Child-centered care; emphasizing the importance of being sensitive and responding to the infant’s cues The meal as a safe base for exploration	Sensitive/Responsive feeding; mother withdraws when the child has mouth closed How to cut and mash fruit and vegetables How to easily make varied baby food
8 months Food with lumps	Introduction to new consistencies and textures Gradual development of the oral-motor skills and mastery of firmer consistencies	Variety in taste and texture and repeated exposure to promote good eating habits Making homemade baby-food to facilitate introduction to the family’s food	Child-centered care; emphasizing the importance of being sensitive and responding to the infant’s cues The meal as a safe base for exploration	Sensitive/Responsive feeding and Family-meal/Modelling; child picking pieces of food to eat Age-appropriate, soft consistencies How to make homemade food with varied consistency suitable for both baby and family
9 months Eating alone and eating together	Age appropriate portion sizes Recognition of hunger and fullness Facilitate self-feeding	How to promote child autonomy and mastery in mealtimes Awareness of the child’s signals of hunger and satiety; parent provide and child decide Avoiding pressure to eat The importance of family-meals and role-modelling Promoting good mealtime routines	Child-centered care; emphasizing the importance of being sensitive and responding to the infant’s cues The meal as a safe base for exploration Parents` responsibility for creating a nurturing emotional climate	Sensitive/Responsive feeding and Family-meal/Modelling; family-dinner with children allowed to explore food on their own How to make homemade food easy for the child to eat, suitable for both baby and family
10 months Food and feelings	Early establishment of food habits and eating behaviors	Avoiding food as comfort or reward Discourage use of restrictive and coercive feeding practices Parents as positive role-models of eating behaviours Promoting good mealtime routines	Child-centered care; emphasizing the importance of being sensitive and responding to the infant’s cues Regulation of emotions; confirm and meet the child’s signals to facilitate self-regulation Role modelling and social referencing; infants read and interpret facial expressions to understand their surroundings	Sensitive/Responsive feeding and Family-meal/Modelling; mother who comforts her child and regulates his feelings Children enjoying eating healthy food How to make homemade food for weekends and celebration, suitable for both baby and family
11 months Food as building blocks – the important foundation	Early food habits and their relevance for later health and prevention of noncommunicable diseases A balanced diet based on readily available raw materials is sufficient and “good enough” Using fruit as a snack, water when thirsty	Variation and repeated exposure to facilitate acceptance of healthy foods like vegetables Making homemade baby-food for greater variety Having fruits and vegetables readily available at home	Child-centered care; emphasizing the importance of being sensitive and responding to the infant’s cues Early, everyday experiences form patterns (cognitive schemas), and lay the foundation for later mental and physical health	Sensitive/Responsive feeding and Family-meal/Modelling; child “helping” her mother to prepare food showing enjoyment and engagement How to make homemade food rich in important nutrients, suitable for both baby and family
12 months Life ahead – weekdays with job and kindergarten	Introduction to family food and appropriate mealtime-structure Using fruit as a snack, water when thirsty	Planning for healthy food-choices Variation and repeated exposure to facilitate acceptance of healthy foods like vegetables Involve the child in preparing meals Avoiding pressure to eat; parent provide, child decide Promoting good mealtime routines Parents and other children as positive role-models of eating behaviours	Child-centered care; emphasizing the importance of being sensitive and responding to the infant’s cues The meal as a safe base for exploration Parents` responsibility for creating a nurturing emotional climate	Sensitive/Responsive feeding and Family-meal/Modelling; child allowed to assist in preparing the meal How to make homemade food for busy weekdays, suitable for both baby and family

### Measurement and instruments

Outcome variables to be measured are listed in Table 3. Most of the instruments are previously validated and have been used in the same age groups as intended in this study in Norway or other countries.

**Table 3:** Description of variables, purpose of measure, instruments, and when data will be collected

Level	Purpose of measure	Variable	Measure	Instrument	When to collect
Infant	SO	Antropo-metric measures	Weight and height	Self-reported, but measured at the scheduled visits to the health centre	At 6 and 12 months (24 and 48 months^a^)
	SO, IC	Food intake Food variance	Daily food intake, breastfeeding, introduction of solid food	FFQ based on FFQ from Norwegian nationwide diet surveys among 6 and 12 months old children [13, 56] and the Norwegian MoBa-study [51]	At 6 and 12 months (24 and 48 months^a^)
	SO, IC	Child eating behaviour	Report of satiety-responsiveness, fussiness, food enjoyment, emotional over−/under eating	BEBQ [57, 58] CEBQ [59, 60] CEBQ	At 6 months At 12 months (24 and 48 months^a^)
	SC	Infant tempera-ment	Infant difficultness as perceived by caregiver	Fussy/difficult subscale of the Infant Characteristics Questionnaire [61, 62]	At 6 and 12 months
	SO, IC	Food preferences	Rating of child’s preferences for listed food and beverages	Food preferences questionnaire developed for this study’s purpose	(At 24 and 48 months^a^)
	SO, SC	Child behaviour	Internalizing and externalizing behaviours	Child Behaviour CheckList (CBCL) [63–65]	(At 24 and 48 months^a^)
Parent	SC	Antropo-metric measures	Weight and height of parent	Self-reported	At 6 and 12 months (24 and 48 months^a^)
	SC	Food intake Food variance	Frequency of intake of indicator foods for healthy and unhealthy food.	FFQ developed for this study’s purpose, based on the Norwegian MoBa-study [51]	At 6 and 12 months (24 and 48 months^a^)
	SO, IC	Food neophobia	Ratings of the parent’s courage to taste new foods and flavours	The food neophobia scale [66]	At 6 months
	SC	Parental eating behaviour	Report of satiety-responsiveness, fussiness, food enjoyment, emotional over−/under eating	Adult Eating Behaviour Questionnaire [67] (AEBQ)	At 12 months (24 and 48 months^a^)
	SO, IC	Feeding style and feeding practices	Feeding attitudes, practices, perceptions/concern regarding weight, under/over-eating, infant cues	Infant Feeding Questionnaire [68, 69] Child Feeding Questionnaire [70]	At 6 and 12 months (24 and 48 months^a^)
	SO, IC	Feeding self efficacy	Parental self- efficacy in feeding situations	Five items from the Feeding Self-Efficacy Questionnaire [71, 72]	At 6 and 12 months (24 and 48 months^a^)
	SO, IC	Parenting style	Parental discipline, routine, anxiety, nurturance and involvement	Infancy Parenting Styles Questionnaire [73]	At 6 and 12 months
	SO, IC	Parenting style	Control-oriented parenting	The Parental Locus of Control Scale (PLOC), short version [74, 75]	At 12 months (24 and 48 months^a^)
	SC	Parental personality traits and mental health	General self-efficacy Negative Affectivity	Short versions of: General Self-Efficacy Scale (GSE) [76, 77] The (Hopkins) Symptoms Checklist (SCL-8) [76, 78, 79] Edinburgh Postnatal Depression Scale (EPDS) [80, 81]	At 6 and 12 months (24 and 48 months^a^) At 12 months

*SO* Study outcome, *IC* Intervention component, *SC* Study covariate, *FFQ* Food frequency questionnaire, *BEBQ* Baby Eating Behaviour Questionnaire, *CEBQ* Child Eating Behaviour Questionnaire

^a^Possible follow-up studies, not yet planned

### User involvement and process-evaluation

As a prelude to the development of the intervention, a group of public child health nurses in a municipality were interviewed regarding their perceptions and experiences with nutritional guidance to parents of infants and toddlers. Since Norwegian children are followed regularly by the municipal health centers, the public child health nurses are important stakeholders when it comes to advice and guidance on infant nutrition. The public child health nurses also constitute a significant user group. Their perception of an intervention’s usability is crucial to whether they recommend it to their parents. The public child health nurses provided valuable key insights that were used in the development of the intervention.

After developing the script for the intervention’s theme-films, a group of seven parents gave feedback on the scripts’ content in terms of how well it matched their child’s age, if the text was easily understood and if the content was perceived as important and relevant.

To evaluate the intervention, we have also included questions for parents in the intervention group about their experiences in the questionnaire at T2 (12 months). In addition, a Master student has conducted qualitative in-depth interviews with seven parents in the intervention group about their expectations and the perceived usefulness of the intervention.

### Sample size calculations

Daniels et al. in the NOURISH study protocol [7] estimated that 265 participants per group would be a sufficient sample size to be able to detect differences in feeding practices with 80% power and type error I of 5%. Their observed differences in weight measures did not, however, reach statistical significance [17]. Taking this into account, we estimated that we needed 400 participants in both control and intervention arm (800 in total) and aimed to recruit 500 in each group assuming a completion rate of 80%.

### Data analyses

Data have not yet been analysed. To detect potential differences in study outcomes between the intervention and control groups, between-group comparisons and multivariate analyses will be done.

## Discussion

The completion rate at baseline was 75%, slightly lower than what we assumed. Parents who wanted to participate in the study could be enrolled when the child was 3 months old, but the questionnaire prior to randomization and intervention were to be submitted when the child was between five and 6 months old. The time span between study recruitment and the baseline questionnaire may explain the drop-offs of participants from recruitment time to inclusion and randomization.

Although both mothers and fathers were invited to participate in the study, a large majority of the recruited participants were mothers. It may be that more mothers than fathers are frequent users of Facebook, and that this way of recruitment is not as suitable for men as for women. However, according to Plantin & Daneback, parent’s online behaviour reflects their offline behaviour when it comes to searching for information about health and parenting at the Internet. That mothers more frequently than fathers are searching for health-related information on the web, may reflect that women most often take the main responsibility for the hands-on healthcare of the family [23, 49]. This corresponds with other research in this area, which until now has been dominated by research on mothers and children [31].

The educational level in the participant group was relatively high, which may raise questions about selection bias and limited generalizability of results. This skewness is well known from epidemiological research. Lower participation and response rates in groups from less advantaged socio-economic classes have been a major concern in many population-based health surveys [50]. This is also seen in the large Norwegian Mother and Child cohort study (MoBa) [51], where the participating mothers had healthier lifestyle patterns and higher socio-economic status than the average population [52, 53]. Two studies comparing MoBa participant’s data with non-participant’s data obtained from national registry-data, found that differences between the groups had implications for prevalence studies but did not appear to compromise exposure-outcome associations [53, 54].

In this study, we anticipated that using advertising on Facebook as recruitment method and an easy accessible, online study design would make it easier for parents from lower socioeconomic levels to participate. This does not seem to be the case, and raises the question if use of Internet-based strategies could be even more prone to selection bias. Few studies have investigated this, but a recent Danish study evaluated the occurrence of selection bias in an Internet-based study of pregnancy planners using external data from the Danish Medical Birth Registry. They found that recruiting reproductive aged women through the Internet did not led to more selection bias than traditional methods of recruitment [55].

## Conclusion

The early living environment is important for children’s development and may lay the foundation for lifelong patterns. This also applies to the establishment of early eating habits. Providing parents with guidance on diet and nutrition during this significant period is an important task for the health authorities and public child health services. Parents, especially mothers, are extensive users of the Internet to seek for health-related information regarding their children. Despite this, there are few randomized controlled studies investigating the use of Internet to promote healthy eating habits in early childhood, which may prevent childhood overweight and obesity and lay a good foundation for future health.

The eHealth intervention in *Early Food for Future Health* guides parents of children between 6 and 12 months of age through food-related developmental stages in the weaning period. It is an intervention with focus on promoting early healthy food-habits suitable for all children and parents. The size of the study, the web-based design and the comprehensive mapping of possible modifying factors may provide greater insight and understanding of the important field of preventing childhood overweight and obesity on following topics: Design and efficacy of internet-based interventions and the complex and reciprocal relationship between parenting, feeding behavior and children’s eating behavior.

## References

[1] Wang Y, Lobstein T (2006). Worldwide trends in childhood overweight and obesity. Int J Pediatr Obes.

[2] de Onis M, Blossner M, Borghi E (2010). Global prevalence and trends of overweight and obesity among preschool children. Am J Clin Nutr.

[3] Strauss RS, Pollack HA (2003). Social marginalization of overweight children. Arch Pediatr Adolesc Med..

[4] Reilly JJ, Kelly J (2011). Long-term impact of overweight and obesity in childhood and adolescence on morbidity and premature mortality in adulthood: systematic review. Int J Obes.

[5] O'Neil A (2014). Relationship between diet and mental health in children and adolescents: a systematic review. Am J Public Health.

[6] Norwegian Institute of Public Health (2010). The child growth study in Norway.

[7] Daniels LA (2009). The NOURISH randomised control trial: positive feeding practices and food preferences in early childhood - a primary prevention program for childhood obesity. BMC Public Health.

[8] Rodgers RF (2013). Maternal feeding practices predict weight gain and obesogenic eating behaviors in young children: a prospective study. Int J Behav Nutri Phys Act..

[9] Chaput JP (2011). Modern sedentary activities promote overconsumption of food in our current obesogenic environment. Obes Res..

[10] Ong KK, Loos RJ (2006). Rapid infancy weight gain and subsequent obesity: systematic reviews and hopeful suggestions. Acta Paediatr.

[11] Musher-Eizenman DR, Kiefner A (2013). Food parenting: a selective review of current measurement and an empirical examination to inform future measurement. Childhood Obesity (Print).

[12] Norwegian Health Directorate (2017). Nasjonal faglig retningslinje for det helsefremmende og forebyggende arbeidet i helsestasjon IS-2582.

[13] Norwegian Health Directorate (2008). Spedkost 6 months: Nationwide diet survey among 6 months old children.

[14] Birch LL, Fisher JO (1998). Development of eating behaviors among children and adolescents. Pediatrics.

[15] Mallan KM (2014). Satiety responsiveness in toddlerhood predicts energy intake and weight status at four years of age. Appetite.

[16] Cooke L (2007). The importance of exposure for healthy eating in childhood: a review. J Hum Nutr Diet..

[17] Daniels LA (2013). Outcomes of an early feeding practices intervention to prevent childhood obesity. Pediatrics.

[18] Paul IM (2011). Preventing obesity during infancy: a pilot study. Obesity (Silver Spring, Md).

[19] Redsell SA (2015). Systematic review of randomised controlled trials of interventions that aim to reduce the risk, either directly or indirectly, of overweight and obesity in infancy and early childhood. Matern Child Nutr.

[20] Daniels LA (2015). An early feeding practices intervention for obesity prevention. Pediatrics.

[21] Bert F (2013). Pregnancy e-health: a multicenter Italian cross-sectional study on internet use and decision-making among pregnant women. J Epidemiol Community Health.

[22] Eysenbach G (2001). What is e-health?. J Med Internet Res.

[23] Plantin L, Daneback K (2009). Parenthood, information and support on the internet. A literature review of research on parents and professionals online. BMC Fam Pract.

[24] White M, Dorman SM (2001). Receiving social support online: implications for health education. Health Educ Res.

[25] Salonen AH (2014). Impact of an internet-based intervention on Finnish mothers' perceptions of parenting satisfaction, infant centrality and depressive symptoms during the postpartum year. Midwifery.

[26] Park E, Kim H, Steinhoff A (2016). Health-related internet use by informal caregivers of children and adolescents: an integrative literature review. J Med Internet Res.

[27] Uesugi KH (2016). Design of a Digital-Based, multicomponent nutrition guidance system for prevention of early childhood obesity. J Obes.

[28] Ventura AK, Birch LL (2008). Does parenting affect children's eating and weight status?. Int J Behav Nutr Phys Act.

[29] Hetherington MM (2011). Feeding infants and young children. From guidelines to practice. Appetite.

[30] Daniels LA (2012). Evaluation of an intervention to promote protective infant feeding practices to prevent childhood obesity: outcomes of the NOURISH RCT at 14 months of age and 6 months post the first of two intervention modules. Int J Obes.

[31] Jansen E, Daniels LA, Nicholson JM (2012). The dynamics of parenting and early feeding--constructs and controversies: a viewpoint. Early Child Dev Care.

[32] Darling N, Steinberg L (1993). Parenting style as context - an integrative model. Psychol Bull.

[33] Gerards SM, Kremers SP (2015). The role of food parenting skills and the home food environment in Children's weight gain and obesity. Curr Obes Rep.

[34] Bowlby J (1982). Attachment and loss: retrospect and prospect. Am J Orthop.

[35] Ainsworth MD, Bell SM (1970). Attachment, exploration, and separation: illustrated by the behavior of one-year-olds in a strange situation. Child Dev.

[36] Brumariu LE, Kerns KA (2010). Parent-child attachment and internalizing symptoms in childhood and adolescence: a review of empirical findings and future directions. Dev Psychopathol.

[37] Pinquart M (2014). Associations of general parenting and parent-child relationship with pediatric obesity: a meta-analysis. J Pediatr Psychol.

[38] Letourneau N (2015). Narrative and meta-analytic review of interventions aiming to improve maternal-child attachment security. Infant Ment Health J.

[39] Bakermans-Kranenburg MJ, van Ijzendoorn MH, Juffer F (2003). Less is more: meta-analyses of sensitivity and attachment interventions in early childhood. Psychol Bull.

[40] Bandura A (2004). Health promotion by social cognitive means. Health Educ Behav.

[41] Campbell KJ (2013). A parent-focused intervention to reduce infant obesity risk behaviors: a randomized trial. Pediatrics.

[42] Cameron AJ (2015). A review of the relationship between socioeconomic position and the early-life predictors of obesity. Curr Obes Rep.

[43] Campbell K (2008). The infant feeding activity and nutrition trial (INFANT) an early intervention to prevent childhood obesity: cluster-randomised controlled trial. BMC Public Health.

[44] Burlingame BA, Dernini S. Sustainable diets and biodiversity: directions and solutions for policy, research and action, in Sustainable diets and biodiversity: directions and solutions for policy, research and action. Rome: FAO: 2012.

[45] Hellen C. Tidsskrift for Helsesøstre, 2016. nr. 1-2016: p. 40-42.

[46] University of Agder (2016). Early food for future health.

[47] Schwartz C (2011). Development of healthy eating habits early in life. Review of recent evidence and selected guidelines. Appetite.

[48] Birch LL, Ventura AK (2009). Preventing childhood obesity: what works?. Int J Obes.

[49] Daneback K, Plantin L (2008). Research on parenthood and the internet: themes and trends. Cyberpsychology.

[50] Søgaard AJ (2004). The Oslo health study: the impact of self-selection in a large, population-based survey. Int J Equity Health.

[51] Norwegian Institute of Public Health. The Norwegian Mother and Child Cohort Study (MoBa). 2017;1998–2008. Available from: https://www.fhi.no/en/studies/moba/.

[52] Magnus P (2006). Cohort profile: the Norwegian mother and child cohort study (MoBa). Int J Epidemiol.

[53] Nilsen RM (2009). Self-selection and bias in a large prospective pregnancy cohort in Norway. Paediatr Perinat Epidemiol.

[54] Nilsen RM (2013). Analysis of self-selection bias in a population-based cohort study of autism spectrum disorders. Paediatr Perinat Epidemiol.

[55] Hatch EE (2016). Evaluation of selection bias in an internet-based study of pregnancy planners. Epidemiology.

[56] Norweigan Health Directorate (2009). Spedkost 12 months: Nationwide diet survey among 12 months old children.

[57] Llewellyn CH (2011). Development and factor structure of the baby eating behaviour questionnaire in the Gemini birth cohort. Appetite.

[58] Mallan KM, Daniels LA, de Jersey SJ (2014). Confirmatory factor analysis of the baby eating behaviour questionnaire and associations with infant weight, gender and feeding mode in an Australian sample. Appetite.

[59] Carnell S, Wardle J (2007). Measuring behavioural susceptibility to obesity: validation of the child eating behaviour questionnaire. Appetite.

[60] Wardle J (2001). Development of the Children's eating behaviour questionnaire. J Child Psychol Psychiatry.

[61] Niegel S, Ystrom E, Vollrath ME (2007). Is difficult temperament related to overweight and rapid early weight gain in infants? A prospective cohort study. J Dev Behav Pediatr.

[62] Bates JE, Freeland CA, Lounsbury ML (1979). Measurement of infant difficultness. Child Dev.

[63] Achenbach TM. Manual for the child behaviour Checklist/2–3 and 1992 profile. Burlington: University of Vermont Department of Psychiatry. p. 1992.

[64] Novik TS (1999). Validity of the child behaviour Checklist in a Norwegian sample. Eur Child Adolesc Psychiatry.

[65] Jacka FN (2013). Maternal and early postnatal nutrition and mental health of offspring by age 5 years: a prospective cohort study. J Am Acad Child Adolesc Psychiatry.

[66] Pliner P, Hobden K (1992). Development of a scale to measure the trait of food neophobia in humans. Appetite.

[67] Hunot C (2016). Appetitive traits and relationships with BMI in adults: development of the adult eating behaviour questionnaire. Appetite.

[68] Baughcum AE (2001). Maternal feeding practices and beliefs and their relationships to overweight in early childhood. J Dev Behav Pediatr..

[69] Mallan KM (2016). The relationship between maternal feeding beliefs and practices and perceptions of infant eating behaviours at 4 months. Appetite.

[70] Birch LL (2001). Confirmatory factor analysis of the child feeding questionnaire: a measure of parental attitudes, beliefs and practices about child feeding and obesity proneness. Appetite.

[71] Horodynski MA, Stommel M (2005). Nutrition education aimed at toddlers: an intervention study. Pediatr Nurs.

[72] Koh GA (2014). Maternal feeding self-efficacy and fruit and vegetable intakes in infants. Results from the SAIDI study Appetite.

[73] Arnott B, Brown A (2013). An exploration of parenting Behaviours and attitudes during early infancy: association with maternal and infant characteristics. Infant Child Dev.

[74] Campis LK, Lyman RD, Prentice-Dunn S (1986). The parental locus of control scale: development and validation. J Clin Child Psychol..

[75] Ystrom E, Barker M, Vollrath ME (2012). Impact of mothers' negative affectivity, parental locus of control and child-feeding practices on dietary patterns of 3-year-old children: the MoBa cohort study. Matern Child Nutr.

[76] Tambs K, Røysamb E (2014). Selection of questions to short-form versions of original psychometric instruments in MoBa. Norsk Epidemiologi.

[77] Ystrom E (2008). The impact of maternal negative affectivity and general self-efficacy on breastfeeding: the Norwegian mother and child cohort study. J Pediatr.

[78] Derogatis LR, Lipman RS, Covi L (1973). SCL-90: an outpatient psychiatric rating scale--preliminary report. Psychopharmacol Bull.

[79] Ystrom E, Niegel S, Vollrath ME (2009). The impact of maternal negative affectivity on dietary patterns of 18-month-old children in the Norwegian mother and child cohort study. Matern Child Nutr.

[80] Cox JL, Holden JM, Sagovsky R. Detection of postnatal depression. Development of the 10-item Edinburgh Postnatal Depression Scale. Br J Psychiatry. 1987;150:782–6. 10.1192/bjp.150.6.782 3651732

[81] Eberhard-Gran M (2007). A short matrix-version of the Edinburgh depression scale. Acta Psychiatr Scand.

